# Supplementary material for: Mild Zika Virus Infection in Mice Without Motor Impairments Induces Working Memory Deficits, Anxiety-like Behaviors, and Dysregulation of Immunity and Synaptic Vesicle Pathways
Source: Viruses. 2025 Mar 12;17(3):405. doi: 10.3390/v17030405 (PMC11946058; doi:10.3390/v17030405)
Supplement: Supplementary file 1 [file viruses-17-00405-s001.zip › Figure S1. Weight trajectory of infected and control animals over time..pdf]

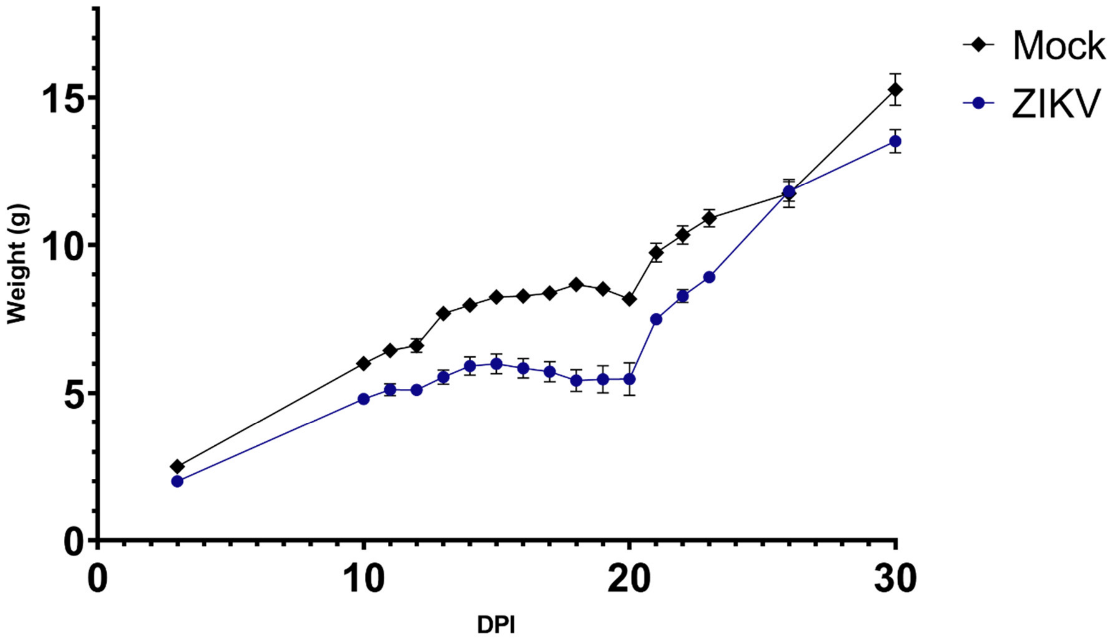

Figure S1. Weight trajectory of infected and control animals over time. The graph depicts body weight (grams) on the Y-axis as a function of days post-infection (DPI) on the X-axis. Black lines represent the control group, while blue lines correspond to the infected group. Error bars indicate the standard error of the mean for each time point.
