# Supplementary material for: Mild Zika Virus Infection in Mice Without Motor Impairments Induces Working Memory Deficits, Anxiety-like Behaviors, and Dysregulation of Immunity and Synaptic Vesicle Pathways
Source: Viruses. 2025 Mar 12;17(3):405. doi: 10.3390/v17030405 (PMC11946058; doi:10.3390/v17030405)
Supplement: Supplementary file 1 [file viruses-17-00405-s001.zip › Figure S2. GO Functional Annotation in Cortex and Cerebellum..pdf]

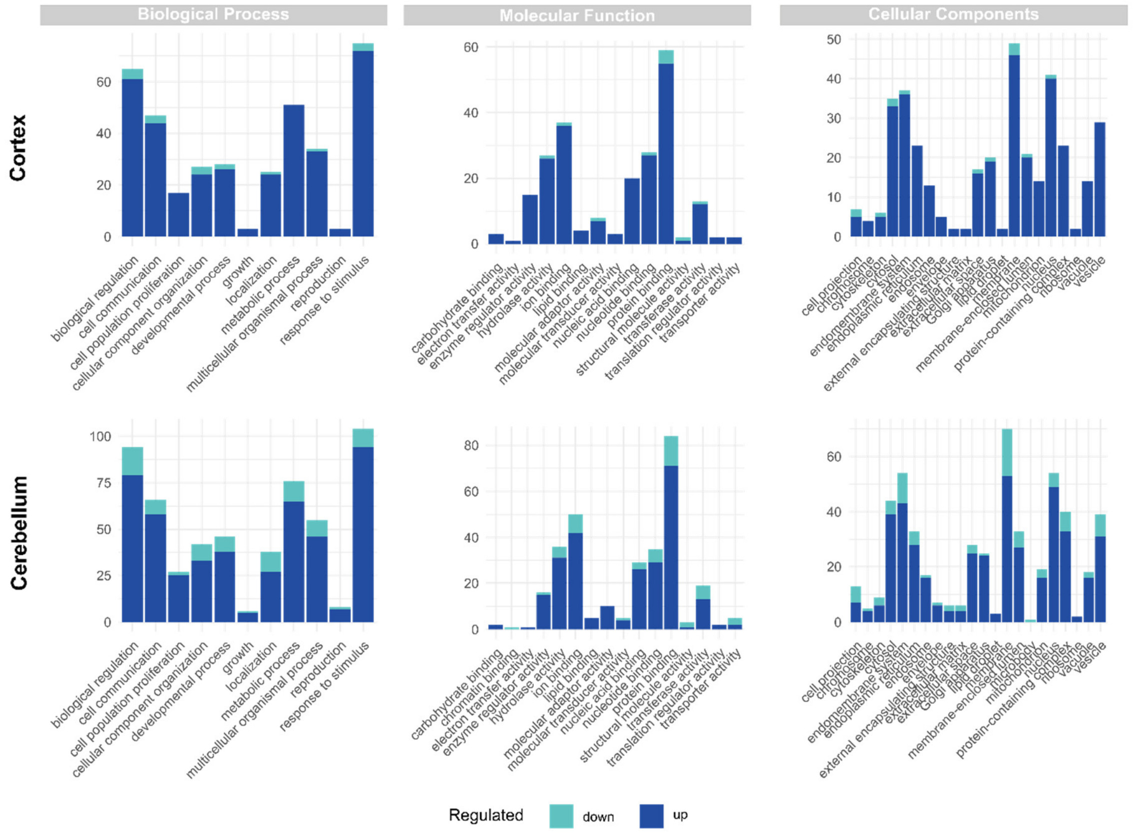

Figure S2. GO Functional Annotation in Cortex and Cerebellum. The categories of Biological Process, Cellular Component, and Molecular Function are represented by differentially expressed genes (DEGs), with upregulated genes shown in blue and downregulated genes in light blue. The height of each bar corresponds to the number of DEGs associated with each function.
