# Supplementary material for: Mild Zika Virus Infection in Mice Without Motor Impairments Induces Working Memory Deficits, Anxiety-like Behaviors, and Dysregulation of Immunity and Synaptic Vesicle Pathways
Source: Viruses. 2025 Mar 12;17(3):405. doi: 10.3390/v17030405 (PMC11946058; doi:10.3390/v17030405)
Supplement: Supplementary file 1 [file viruses-17-00405-s001.zip › Figure S3. Interaction Network of the top DEGs upregulated by Cortex and cerebellum..pdf]

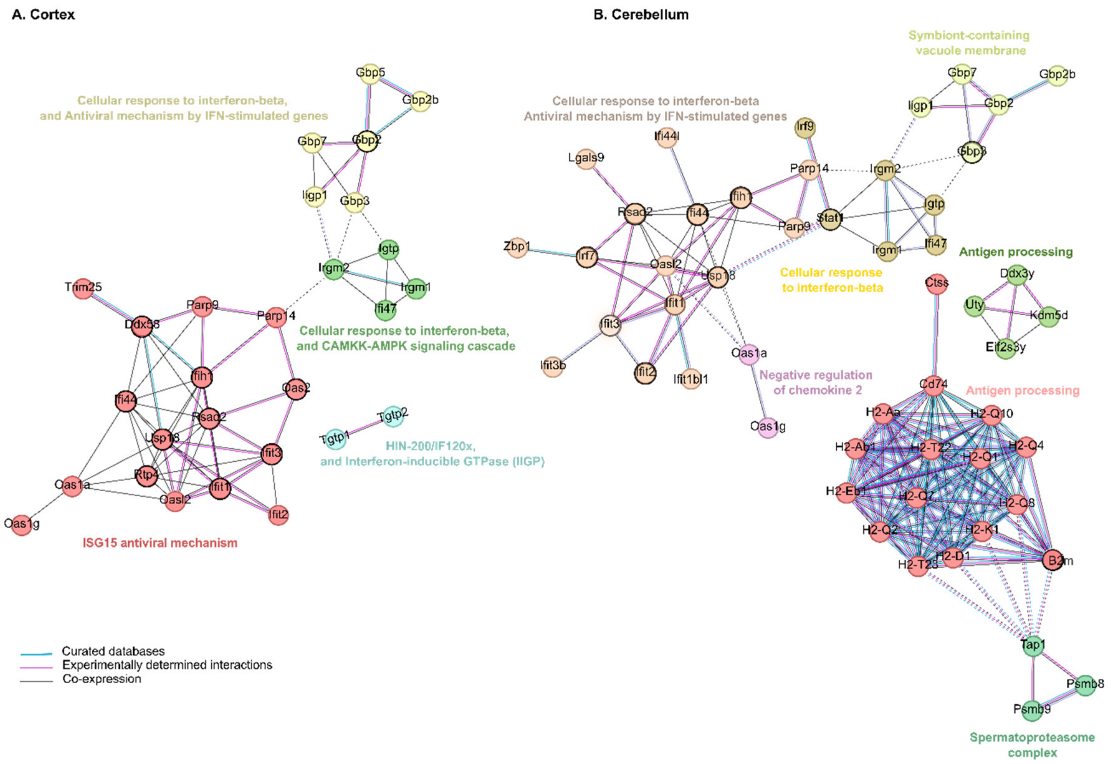

Figure S3. Interaction Network of the top DEGs upregulated by Cortex and cerebellum. Interaction networks with threshold of interaction score >0.7 (high confidence). Nodes are grouped by MCL clustering. Experimentally determined interactions are indicated by pink lines, interactions with curated databases (Reactome, KEGG, etc.) are indicated by blue lines, and black lines indicate co-expression. Solid lines indicate interactions within clusters, and dotted lines indicate interactions between clusters. Networks hub genes are outlined in black. **(A).** DEGs upregulated selected in the cortex. **(B).** DEGs upregulated selected in the cerebellum.
