# Supplementary material for: Mild Zika Virus Infection in Mice Without Motor Impairments Induces Working Memory Deficits, Anxiety-like Behaviors, and Dysregulation of Immunity and Synaptic Vesicle Pathways
Source: Viruses. 2025 Mar 12;17(3):405. doi: 10.3390/v17030405 (PMC11946058; doi:10.3390/v17030405)
Supplement: Supplementary file 1 [file viruses-17-00405-s001.zip › Table S1. Differentially Expressed Genes (DEGs) identified in the cerebellum and cortex..pdf]

Table S1. Differentially Expressed Genes (DEGs) identified in the cerebellum and cortex.

Gene	Log2FoldChange	Padj	Regulated	Tissue
Eif2s3y	900.533.435.147.466	3,83E-07	upregulated	Cerebellum
Uty	800.447.791.091.851	7,52E-27	upregulated	Cerebellum
Kdm5d	770.331.213.727.597	1,24E-03	upregulated	Cerebellum
Tgtp2	603.343.842.139.709	2,09E-01	upregulated	Cerebellum
Igha	545.723.712.263.122	1,82E+14	upregulated	Cerebellum
Iigp1	509.567.766.111.398	4,31E-02	upregulated	Cerebellum
Ighg2c	505.754.566.841.023	4,81E+13	upregulated	Cerebellum
Ighg2b	502.749.166.455.443	4,11E+14	upregulated	Cerebellum
Igtp	497.799.383.897.733	2,72E+03	upregulated	Cerebellum
Ifi44l	491.411.607.424.992	7,05E+14	upregulated	Cerebellum
Iigp1c	491.348.382.886.106	3,14E-01	upregulated	Cerebellum
H2-Aa	481.714.684.863.488	1,25E+08	upregulated	Cerebellum
Ddx3y	478.433.078.378.724	6,05E-01	upregulated	Cerebellum
Zbp1	469.814.352.906.588	6,29E+14	upregulated	Cerebellum
F830016B08Rik	465.148.757.355.872	1,26E+04	upregulated	Cerebellum
Ifit1	454.986.319.469.833	2,22E-02	upregulated	Cerebellum
Oas1a	448.819.350.773.005	9,05E+09	upregulated	Cerebellum
Igkc	447.812.364.932.517	1,56E+05	upregulated	Cerebellum
Tgtp1	440.168.329.013.671	1,28E+08	upregulated	Cerebellum
Mmp12	425.336.616.281.221	3,85E+14	upregulated	Cerebellum
Oas1g	424.526.011.377.508	9,95E+14	upregulated	Cerebellum
Nlrc5	423.991.075.372.424	6,16E+04	upregulated	Cerebellum
Gm10499	421.639.132.560.382	4,19E+04	upregulated	Cerebellum
Gm42074	411.749.838.690.728	4,24E+06	upregulated	Cerebellum
Ifi47	411.303.991.985.838	2,91E+14	upregulated	Cerebellum
Ifi207	407.488.641.441.375	9,84E+12	upregulated	Cerebellum
H2-T27	403.123.548.162.861	6,35E+09	upregulated	Cerebellum
Gm31785	400.064.131.998.394	6,16E+04	upregulated	Cerebellum
H2-Q9	397.888.793.701.781	6,22E+13	upregulated	Cerebellum
H2-Q6	395.155.327.237.018	3,06E+07	upregulated	Cerebellum
H2-Q8	392.658.300.832.818	2,49E+08	upregulated	Cerebellum

Gene	Log2FoldChange	Padj	Regulated	Tissue
Gbp10	390.661.675.560.452	1,96E-02	upregulated	Cerebellum
Ifi204	389.981.901.601.474	1,44E+14	upregulated	Cerebellum
Jchain	384.166.471.485.247	2,80E+14	upregulated	Cerebellum
H2-Q7	377.197.086.606.044	1,34E+13	upregulated	Cerebellum
Psmb9	371.310.742.984.418	1,64E+14	upregulated	Cerebellum
Cd74	369.884.546.636.817	4,98E+05	upregulated	Cerebellum
Gbp3	368.788.166.239.141	2,16E+07	upregulated	Cerebellum
Ly6a	365.759.993.206.023	1,56E+07	upregulated	Cerebellum
Gbp6	363.543.839.170.502	1,07E+04	upregulated	Cerebellum
H2-Q1	362.387.330.228.469	7,21E+03	upregulated	Cerebellum
Slfn8	355.997.770.103.483	6,08E+09	upregulated	Cerebellum
B2m	354.486.386.241.252	4,15E-22	upregulated	Cerebellum
H2-D1	354.358.866.152.213	1,56E+00	upregulated	Cerebellum
Ddx60	352.778.577.818.135	3,40E+02	upregulated	Cerebellum
Patl2	350.052.567.864.727	5,20E-25	upregulated	Cerebellum
Mndal	345.916.724.504.445	5,22E+08	upregulated	Cerebellum
H2-Eb1	343.527.676.054.714	3,26E+14	upregulated	Cerebellum
H2-Q4	337.166.579.003.479	9,79E+06	upregulated	Cerebellum
Epsti1	334.198.363.200.239	1,10E+14	upregulated	Cerebellum
Gm54185	333.804.062.602.763	1,50E+14	upregulated	Cerebellum
H2-K1	326.872.011.537.464	2,70E-03	upregulated	Cerebellum
Gbp9	326.143.182.271.922	9,31E+09	upregulated	Cerebellum
Itgax	320.457.925.021.193	4,91E+14	upregulated	Cerebellum
Gbp4	320.306.202.809.576	1,34E+14	upregulated	Cerebellum
H2-Ab1	319.873.442.934.563	5,15E+06	upregulated	Cerebellum
Irgm1	315.016.948.755.406	9,72E+05	upregulated	Cerebellum
Lyz2	314.399.118.741.545	5,52E+14	upregulated	Cerebellum
Ms4a6b	314.365.662.004.242	2,62E+14	upregulated	Cerebellum
Tap1	306.196.694.930.885	2,19E+12	upregulated	Cerebellum
Bst2	302.284.709.165.547	1,80E+14	upregulated	Cerebellum
Ifit1bl1	294.950.622.912.015	1,43E-01	upregulated	Cerebellum

Gene	Log2FoldChange	Padj	Regulated	Tissue
Gm12250	293.017.360.657.081	2,93E+13	upregulated	Cerebellum
Ifit3b	286.339.477.582.595	3,11E+09	upregulated	Cerebellum
Gbp7	283.605.010.302.097	6,89E+08	upregulated	Cerebellum
Gbp2	280.744.267.139.392	4,12E+09	upregulated	Cerebellum
H2-T23	278.812.232.758.566	3,36E+08	upregulated	Cerebellum
Samd9l	275.932.707.422.553	1,09E-01	upregulated	Cerebellum
Cd274	274.191.850.392.858	2,14E+13	upregulated	Cerebellum
Rsad2	271.324.412.906.418	1,71E+14	upregulated	Cerebellum
Cybb	264.489.461.934.236	3,81E+08	upregulated	Cerebellum
Ifi27	262.194.213.134.349	8,19E+06	upregulated	Cerebellum
C3	261.432.986.023.195	4,59E+14	upregulated	Cerebellum
Gm28068	259.719.686.178.118	5,52E+14	upregulated	Cerebellum
Ifi203	258.421.001.700.045	2,24E+13	upregulated	Cerebellum
Ms4a6c	257.879.102.384.338	1,09E+14	upregulated	Cerebellum
Gm44148	256.828.800.759.172	2,21E+14	upregulated	Cerebellum
Gvin2	252.408.765.224.108	1,71E+13	upregulated	Cerebellum
Uba7	251.884.784.928.715	3,50E+14	upregulated	Cerebellum
Themis	251.765.792.675.448	1,40E+14	upregulated	Cerebellum
Gvin1	249.425.548.939.389	6,33E+06	upregulated	Cerebellum
Lgals3bp	247.920.581.191.231	2,37E+08	upregulated	Cerebellum
Il7	246.899.881.494.524	1,93E+14	upregulated	Cerebellum
Gm53496	245.456.678.232.848	3,07E+09	upregulated	Cerebellum
C4b	242.940.460.850.655	3,54E+14	upregulated	Cerebellum
Gm45969	242.395.015.691.661	1,80E+13	upregulated	Cerebellum
Clec7a	235.477.016.683.875	2,91E+14	upregulated	Cerebellum
Stat1	230.654.704.862.212	1,20E+01	upregulated	Cerebellum
Lgals9	226.890.501.008.289	4,19E+14	upregulated	Cerebellum
Parp14	225.320.542.830.371	4,70E+09	upregulated	Cerebellum
Apobec1	224.822.033.584.173	5,22E+08	upregulated	Cerebellum
Xaf1	222.500.212.045.018	2,66E+14	upregulated	Cerebellum
Herc6	222.065.971.661.285	9,79E+06	upregulated	Cerebellum

Gene	Log2FoldChange	Padj	Regulated	Tissue
Gm6034	214.640.302.808.192	2,10E+14	upregulated	Cerebellum
Gm20559	211.362.632.899.884	2,54E+09	upregulated	Cerebellum
Ptprc	210.291.880.453.515	9,78E+08	upregulated	Cerebellum
Fgl2	204.108.901.796.948	3,25E+14	upregulated	Cerebellum
Ifitm3	201.297.256.081.558	7,87E+14	upregulated	Cerebellum
Irf9	197.632.255.944.211	1,71E+13	upregulated	Cerebellum
Ifih1	195.623.439.886.754	2,00E+14	upregulated	Cerebellum
Mpeg1	185.034.233.496.707	5,22E+08	upregulated	Cerebellum
C1qa	182.511.825.064.087	4,28E+09	upregulated	Cerebellum
Dtx3l	179.956.716.398.309	6,95E+09	upregulated	Cerebellum
Cd84	177.506.110.800.877	1,34E+14	upregulated	Cerebellum
Ctss	177.434.882.599.836	1,32E+08	upregulated	Cerebellum
Rnf213	174.443.589.314.324	5,04E+14	upregulated	Cerebellum
A930037H05Rik	173.933.588.875.324	2,35E+14	upregulated	Cerebellum
Parp9	173.313.495.891.208	9,85E+14	upregulated	Cerebellum
Slfn5	171.019.531.890.356	4,62E+12	upregulated	Cerebellum
Ifit2	165.453.809.904.419	4,47E+14	upregulated	Cerebellum
H2-T22	164.557.760.899.069	1,83E+13	upregulated	Cerebellum
Ly86	162.427.873.999.443	3,95E+14	upregulated	Cerebellum
Ighm	159.099.829.644.471	2,03E+14	upregulated	Cerebellum
Fcgr3	158.395.240.509.019	4,90E+13	upregulated	Cerebellum
Arhgap15	149.386.583.899.464	1,88E+14	upregulated	Cerebellum
C1qb	149.345.814.260.894	1,57E+14	upregulated	Cerebellum
Trim34b	149.208.728.770.523	4,55E+14	upregulated	Cerebellum
Gm53283	147.749.174.329.972	2,62E+14	upregulated	Cerebellum
Trim12c	140.186.017.832.151	2,27E+14	upregulated	Cerebellum
Dock2	112.649.398.929.235	8,30E+14	upregulated	Cerebellum
Angpt1	110.939.838.890.079	1,69E+14	upregulated	Cerebellum
Ifi44	49.734.051.522.301	2,64E+04	upregulated	Cerebellum
Irgm2	44.800.660.111.028	2,74E+03	upregulated	Cerebellum
Gm40158	43.019.253.316.144	8,96E+09	upregulated	Cerebellum

Gene	Log2FoldChange	Padj	Regulated	Tissue
Oasl2	40.819.744.669.733	9,98E+08	upregulated	Cerebellum
H2-Q2	39.463.618.582.902	4,78E+06	upregulated	Cerebellum
Gm53999	37.523.186.741.058	4,13E+02	upregulated	Cerebellum
Ifi202b	36.103.971.710.362	5,24E+14	upregulated	Cerebellum
Irf7	34.808.270.345.904	2,29E+13	upregulated	Cerebellum
Psmb8	34.677.005.259.943	9,52E+14	upregulated	Cerebellum
Gbp2b	33.215.861.459.985	2,13E+07	upregulated	Cerebellum
H2-Q10	31.290.106.058.556	2,49E+14	upregulated	Cerebellum
Ifit3	29.987.361.883.587	1,85E+04	upregulated	Cerebellum
Ifi27l2a	28.200.984.161.461	4,09E+04	upregulated	Cerebellum
Usp18	27.394.706.755.589	1,80E+14	upregulated	Cerebellum
Parp10	24.310.209.387.669	1,40E+14	upregulated	Cerebellum
Zc3hav1	22.608.968.431.639	3,15E+04	upregulated	Cerebellum
Rigi	20.275.698.399.734	2,65E+14	upregulated	Cerebellum
9930111J21Rik1	19.416.162.144.654	1,40E+14	upregulated	Cerebellum
Parp12	19.074.701.645.727	7,66E+07	upregulated	Cerebellum
Gm8995	2.568.737.824.131	5,19E+14	upregulated	Cerebellum
Prrt1	-2.701.607.756.457	2,78E+14	dowregulated	Cerebellum
Atp6v1g2	-23.914.845.295.052	4,91E+14	dowregulated	Cerebellum
Copg2os2	-31.868.728.373.121	3,95E+14	dowregulated	Cerebellum
Sspn	-48.219.647.571.048	2,33E+13	dowregulated	Cerebellum
Col6a6	-49.288.475.919.932	4,38E+14	dowregulated	Cerebellum
Xist	-105.601.275.538.016	2,76E-58	dowregulated	Cerebellum
Kit	-115.210.662.581.641	3,13E+14	dowregulated	Cerebellum
Tlcd4	-165.785.960.637.018	2,71E+14	dowregulated	Cerebellum
Pcp2	-197.985.935.660.507	2,93E+13	dowregulated	Cerebellum
Copg2	-222.414.684.394.506	3,33E+14	dowregulated	Cerebellum
Kcnc3	-232.187.867.682.248	2,33E+13	dowregulated	Cerebellum
Mest	-238.093.344.761.431	2,26E+14	dowregulated	Cerebellum
Arl8a	-240.686.697.483.922	4,91E+14	dowregulated	Cerebellum
Prkcg	-247.479.454.794.062	5,37E+14	dowregulated	Cerebellum

Gene	Log2FoldChange	Padj	Regulated	Tissue
mt-Rnr1	-249.130.327.325.998	3,63E+14	dowregulated	Cerebellum
Dao	-255.405.570.758.466	1,52E+14	dowregulated	Cerebellum
Gm15816	-268.596.534.970.756	2,33E+13	dowregulated	Cerebellum
Klc2	-274.682.379.902.314	2,39E+14	dowregulated	Cerebellum
Dtx3	-286.126.459.340.228	3,10E+14	dowregulated	Cerebellum
Fam219aos	-289.221.778.120.335	4,00E+14	dowregulated	Cerebellum
Gm46128	-291.187.513.594.216	4,38E+14	dowregulated	Cerebellum
Mospd3	-293.530.474.932.081	2,33E+13	dowregulated	Cerebellum
Tmem98	-299.314.444.874.887	2,33E+13	dowregulated	Cerebellum
Pabpc1l2b	-302.355.428.347.292	3,39E+14	dowregulated	Cerebellum
Grid2ip	-302.996.751.190.456	2,23E+14	dowregulated	Cerebellum
Phpt1	-307.603.314.269.458	4,08E+14	dowregulated	Cerebellum
Them6	-307.708.929.186.007	2,26E+14	dowregulated	Cerebellum
Slc22a29	-308.811.806.711.223	1,61E+14	dowregulated	Cerebellum
Pabpc1l2a	-311.519.670.372.206	2,33E+13	dowregulated	Cerebellum
Gm30408	-314.914.473.543.143	2,39E+13	dowregulated	Cerebellum
Angptl6	-318.917.918.655.854	2,98E+14	dowregulated	Cerebellum
Hoxb8	-332.557.007.710.132	2,71E+14	dowregulated	Cerebellum
Gm26917	-384.311.929.126.314	3,63E+14	dowregulated	Cerebellum
Zc3h7a	-400.391.168.933.327	1,36E+14	dowregulated	Cerebellum
Gm39429	-416.132.547.395.294	3,63E+14	dowregulated	Cerebellum
Mir6236	-416.141.132.332.666	3,63E+14	dowregulated	Cerebellum
AY036118	-422.089.279.877.541	1,94E+14	dowregulated	Cerebellum
Gm19951	-449.827.265.596.874	3,51E+14	dowregulated	Cerebellum
Gm53756	-449.831.613.130.237	3,51E+14	dowregulated	Cerebellum
Gm36876	-458.622.187.243.512	3,63E+14	dowregulated	Cerebellum
Lars2	-459.843.619.024.394	2,62E+14	dowregulated	Cerebellum
Jarid2	-460.367.645.431.389	3,33E+14	dowregulated	Cerebellum
Tmtc2	-460.776.637.364.889	2,44E+14	dowregulated	Cerebellum
n-R5s102	-463.331.869.696.444	4,11E+13	dowregulated	Cerebellum
Peak1	-496.111.840.831.903	3,60E+13	dowregulated	Cerebellum

Gene	Log2FoldChange	Padj	Regulated	Tissue
n-R5s131	-501.889.106.231.358	1,94E+14	dowregulated	Cerebellum
Gm30821	-518.743.573.630.152	4,59E+14	dowregulated	Cerebellum
Tsix	-948.371.403.957.011	1,74E-86	dowregulated	Cerebellum
Eif2s3y	892.111.827.663.386	7,19E-07	upregulated	Cortex
Kdm5d	782.237.916.239.345	7,66E-06	upregulated	Cortex
Uty	739.835.412.482.691	9,75E-27	upregulated	Cortex
Iigp1	597.320.972.563.939	3,73E+03	upregulated	Cortex
H2-Eb1	585.244.996.506.432	4,28E+08	upregulated	Cortex
Oas1g	580.698.202.654.491	2,13E+13	upregulated	Cortex
Ifi44	568.282.311.611.033	6,11E+05	upregulated	Cortex
Igkc	564.754.478.770.235	8,18E+08	upregulated	Cortex
Iigp1c	537.078.959.549.997	4,74E+01	upregulated	Cortex
Tgtp2	528.839.947.810.773	5,74E+04	upregulated	Cortex
H2-T27	501.338.690.835.711	3,22E+09	upregulated	Cortex
Oas1a	497.851.509.588.328	4,22E+14	upregulated	Cortex
Gm10499	482.592.418.043.625	1,92E+08	upregulated	Cortex
Ddx3y	464.828.058.938.272	2,38E-02	upregulated	Cortex
Mir5107	461.136.735.060.468	4,94E+14	upregulated	Cortex
Gm31785	447.902.219.144.791	4,27E+08	upregulated	Cortex
H2-Q4	439.512.788.973.765	1,81E-03	upregulated	Cortex
Oasl2	439.440.695.243.501	7,01E+08	upregulated	Cortex
Tgtp1	431.811.911.705.472	8,42E+08	upregulated	Cortex
Gm42074	431.515.639.296.014	5,25E+07	upregulated	Cortex
Gbp6	430.574.018.751.065	8,14E+02	upregulated	Cortex
Igtp	428.955.032.346.829	1,59E+07	upregulated	Cortex
Gm12250	427.574.334.348.884	8,34E+14	upregulated	Cortex
Oas2	425.472.938.618.599	2,08E+14	upregulated	Cortex
Gbp3	423.843.473.316.152	3,14E+02	upregulated	Cortex
Cd74	423.332.811.156.146	4,57E+05	upregulated	Cortex
Gbp10	417.596.266.693.403	3,71E+06	upregulated	Cortex
Ifit1	416.911.021.260.572	2,15E+03	upregulated	Cortex

Gene	Log2FoldChange	Padj	Regulated	Tissue
Gm40158	413.213.483.320.483	7,98E+09	upregulated	Cortex
H2-Aa	402.923.594.146.644	3,05E+08	upregulated	Cortex
H2-D1	402.115.671.345.025	1,76E-12	upregulated	Cortex
H2-K1	395.725.908.843.214	4,06E-23	upregulated	Cortex
Siglec1	395.135.013.567.441	1,55E+14	upregulated	Cortex
Gbp4	394.583.401.403.835	3,22E+09	upregulated	Cortex
Mndal	386.762.462.667.363	2,87E+13	upregulated	Cortex
Gbp2b	380.177.458.929.513	1,96E+14	upregulated	Cortex
Nlrc5	372.978.285.658.624	4,82E+06	upregulated	Cortex
H2-Ab1	369.922.324.475.426	2,43E+08	upregulated	Cortex
Gm53999	369.499.699.097.581	3,14E+03	upregulated	Cortex
Ifit3	365.954.688.482.216	6,60E+09	upregulated	Cortex
H2-Q9	364.448.191.723.102	4,85E+14	upregulated	Cortex
Psmb8	360.737.964.926.989	3,50E+12	upregulated	Cortex
Ddx60	358.553.173.181.141	1,61E+02	upregulated	Cortex
Irgm2	357.181.356.656.289	2,43E+09	upregulated	Cortex
Lyz2	356.536.733.711.949	4,19E+14	upregulated	Cortex
Cybb	356.385.609.383.154	2,93E+09	upregulated	Cortex
Ifi203	342.177.379.414.498	2,11E+05	upregulated	Cortex
Irgm1	336.468.560.921.236	4,23E+09	upregulated	Cortex
Xaf1	328.215.394.555.202	2,07E+14	upregulated	Cortex
B2m	326.857.271.638.685	4,92E-07	upregulated	Cortex
Gbp2	324.432.274.236.406	5,15E+14	upregulated	Cortex
Patl2	322.772.355.673.854	1,42E-06	upregulated	Cortex
Usp18	316.516.062.425.891	9,26E+14	upregulated	Cortex
Ly6a	316.154.362.554.677	2,65E+14	upregulated	Cortex
Samd9l	312.712.897.266.179	3,10E+03	upregulated	Cortex
Irf7	312.675.996.826.198	9,52E+14	upregulated	Cortex
Ms4a6b	309.723.695.819.947	4,21E+14	upregulated	Cortex
Gm44148	300.236.237.084.109	1,25E+14	upregulated	Cortex
Gm8995	299.703.603.726.609	2,35E+13	upregulated	Cortex

Gene	Log2FoldChange	Padj	Regulated	Tissue
Ifi27l2a	297.608.576.000.958	4,27E+08	upregulated	Cortex
Tap1	289.715.613.846.643	5,44E+14	upregulated	Cortex
Gvin2	287.724.292.776.546	2,12E+14	upregulated	Cortex
Stat1	285.942.592.003.657	4,08E+00	upregulated	Cortex
Apobec1	284.432.321.171.728	4,94E+08	upregulated	Cortex
Lgals3bp	274.156.765.946.402	1,10E+07	upregulated	Cortex
Ifih1	273.831.909.385.945	5,75E+02	upregulated	Cortex
Uba7	272.849.411.505.118	4,77E+14	upregulated	Cortex
Gvin1	262.559.215.180.123	1,14E+08	upregulated	Cortex
Parp14	256.808.072.822.786	6,60E+09	upregulated	Cortex
Gm20559	254.975.451.630.658	3,75E+14	upregulated	Cortex
C4b	252.062.511.894.221	7,73E+09	upregulated	Cortex
Gbp9	243.638.631.675.793	1,46E+14	upregulated	Cortex
Zc3hav1	230.880.250.169.938	1,01E+08	upregulated	Cortex
C3ar1	230.654.453.487.245	1,17E+14	upregulated	Cortex
Ifitm3	225.820.233.451.161	1,79E+14	upregulated	Cortex
H2-T26	225.695.133.624.817	9,49E+14	upregulated	Cortex
H2-T23	225.092.157.006.084	1,16E+14	upregulated	Cortex
Lgals9	223.051.945.852.796	2,67E+13	upregulated	Cortex
AU020206	217.995.050.649.272	1,57E+09	upregulated	Cortex
Ifit3b	213.297.691.415.824	1,94E+14	upregulated	Cortex
Dtx3l	210.351.808.432.436	1,01E+14	upregulated	Cortex
Irf9	206.941.695.212.501	5,13E+14	upregulated	Cortex
Gm53283	194.345.302.240.555	1,06E+14	upregulated	Cortex
Ptprc	185.783.434.725.575	1,34E+14	upregulated	Cortex
Pik3ap1	181.466.997.158.061	5,99E+14	upregulated	Cortex
Ifit2	177.409.021.301.184	4,77E+09	upregulated	Cortex
Trim25	175.500.356.972.578	7,04E+14	upregulated	Cortex
Parp12	165.796.370.871.874	1,11E+14	upregulated	Cortex
Rnf213	162.803.397.299.537	3,03E+07	upregulated	Cortex
Ctss	154.140.047.999.129	2,41E+13	upregulated	Cortex

Gene	Log2FoldChange	Padj	Regulated	Tissue
Ctsc	151.411.402.147.303	4,29E+14	upregulated	Cortex
H2-T22	131.378.881.814.142	4,95E+14	upregulated	Cortex
Lrmda	117.474.455.545.381	1,45E+14	upregulated	Cortex
Dock2	109.107.330.018.268	7,19E+14	upregulated	Cortex
F830016B08Rik	56.451.673.667.098	6,40E+04	upregulated	Cortex
C3	55.477.105.834.408	2,65E+09	upregulated	Cortex
H2-Q8	43.518.114.988.655	4,77E+09	upregulated	Cortex
H2-Q6	42.295.239.345.728	2,35E+13	upregulated	Cortex
Ms4a6c	39.801.728.057.649	7,31E+14	upregulated	Cortex
Slfn8	33.632.455.402.115	1,28E+09	upregulated	Cortex
Ifi27	28.277.448.198.418	2,43E+09	upregulated	Cortex
H2-Q1	27.560.488.739.016	5,71E+09	upregulated	Cortex
Gbp7	27.052.988.786.917	2,86E+06	upregulated	Cortex
Bst2	26.802.318.043.633	1,32E+14	upregulated	Cortex
Ifit1bl1	23.548.642.760.781	1,82E+02	upregulated	Cortex
Parp9	21.887.406.200.871	5,91E+14	upregulated	Cortex
Slfn5	21.473.488.165.335	9,86E+14	upregulated	Cortex
Mpeg1	19.853.739.828.718	1,52E+09	upregulated	Cortex
Herc6	15.661.485.592.906	4,27E+08	upregulated	Cortex
Shank1	-193.294.757.247.009	4,14E+14	dowregulated	Cortex
Cxcl14	-262.709.349.887.158	4,77E+14	dowregulated	Cortex
Gm16582	-266.331.843.608.619	4,21E+14	dowregulated	Cortex
Gm52861	-269.470.580.628.084	4,95E+14	dowregulated	Cortex
Asphd1	-288.056.585.193.831	2,68E+14	dowregulated	Cortex
Gm52481	-305.952.825.826.421	2,68E+14	dowregulated	Cortex
Phpt1	-310.647.498.201.063	1,94E+14	dowregulated	Cortex
Gm52217	-401.735.108.432.352	6,71E+12	dowregulated	Cortex
Peak1	-419.170.393.359.273	4,14E+14	dowregulated	Cortex
Tsix	-888.288.558.542.806	2,53E-104	dowregulated	Cortex
Xist	-951.521.796.099.311	1,37E-79	dowregulated	Cortex
